# Small molecule targeting amyloid fibrils inhibits *Streptococcus mutans* biofilm formation

**DOI:** 10.1186/s13568-021-01333-2

**Published:** 2021-12-17

**Authors:** Yuanyuan Chen, Guxin Cui, Yuqi Cui, Dongru Chen, Huancai Lin

**Affiliations:** 1 Department of Preventive Dentistry, Hospital of Stomatology, Sun Yat-Sen University, Guangzhou, Guangdong China; 2 Department of Orthodontics, Hospital of Stomatology, Sun Yat-Sen University, Guangzhou, Guangdong China; 3 Guangdong Provincial Key Laboratory of Stomatology, Guanghua School of Stomatology, Sun Yat-Sen University, Guangzhou, Guangdong China

**Keywords:** Amyloid fibrils, *Streptococcus mutans*, Biofilm formation, Small molecule

## Abstract

Amyloid fibrils are important scaffold in bacterial biofilms. *Streptococcus mutans* is an established cariogenic bacteria dwelling within biofilms, and C123 segment of P1 protein is known to form amyloid fibrils in *S. mutans* biofilms, among which C3 segment could serve as a promising anti-amyloid target due to its critical role in C123-P1 interactions. Recently, small molecules have been found to successfully inhibit biofilms by targeting amyloid fibrils. Thus, our study aimed to screen small molecules targeting C3 segment with the capacity to influence amyloid fibrils and *S. mutans* biofilms. In silico screening was utilized to discover promising small molecules, which were evaluated for their effects on bacterial cells and amyloid fibrils. We selected 99 small molecules and enrolled 55 small molecules named D1–D55 for crystal violet staining. Notably, D25 selectively inhibit *S. mutans* biofilms but had no significant influence on biofilms formed by *Streptococcus gordonii* and *Streptococcus sanguinis*, and D25 showed no bactericidal effects and low cytotoxicity. In addition, amyloid fibrils in free-floating bacteria, biofilms and purified C123 were quantified with ThT assays, and the differences were not statistically significant in the presence or absence of D25. Morphological changes of amyloid fibrils were visualized with TEM images, where amorphous aggregates were obvious coupled with long and atypical amyloid fibrils. Moreover, amyloid-related genes were upregulated in response to D25. In conclusion, D25 is a promising antimicrobial agent with the capacity to influence amyloid fibrils and inhibit *S. mutans* biofilms.

**Supplementary Information:**

The online version contains supplementary material available at 10.1186/s13568-021-01333-2.

## Introduction

Biofilms are complex microbial aggregates that are widely distributed in diverse environment (Flemming and Wingender 2010). In robust biofilms, bacterial cells show enhanced resistance to antibiotics, environmental insults and host defenses than free-floating bacterial cells (Costerton et al. 1999; Flemming and Wingender 2010). As a consequence, biofilms are difficult to eradicate and would result in chronic infections. Thus, it is of great importance to find antibiofilm agents that can prevent biofilm formation and ultimately reduce biofilm infections.

Multiple determinants involve in the development and formation of bacterial biofilms, and they vary in different species and environmental conditions (Cegelski et al. 2009; Romero et al. 2010). Nevertheless, polysaccharide, proteins and extracellular DNA are commonly occurring parts, and extracellular polysaccharide have been well-studied for its crucial role affecting pathogenesis (Cegelski et al. 2009; Romero et al. 2010; Bowen and Koo 2011). Recently, another important matrix component variously known as amyloid fibrils seems to be attractive, and has been reported in numerous studies that involves in the biofilm formation of many bacterial species (Larsen et al. 2007). The foremost studied amyloid fibrils are the curli fibers in *Escherichia coli* and *Salmonella* species, which biophysically and biochemically polymerize into pathogenic amyloids and participate in biofilm formation (Chapman et al. 2002; Saldaña et al. 2009; Erskine et al. 2018). Emerging studies on phenol-soluble modulins (PSMs) in *Staphylococcus aureus* and TasA fibers in *Bacillus subtilis* also reveal the importance of amyloid fibrils in biofilm formation (Erskine et al. 2018). The general hallmark of amyloid fibrils in biofilms is their role as protein scaffold to mediate interactions between bacteria and extracellular matrix or interactions with other extracellular components (Jong et al. 2009; Blanco et al. 2012; Loquet et al. 2018). Moreover, they benefit in adhesion to surface and changing surface properties as well as stabilizing the biofilms (Blanco et al. 2012). Due to the vital role of amyloid fibrils in biofilm development, amyloid inhibitors designed have been intensively developed and reported. For instance, α-sheet peptides are potential inhibitors for *S. aureus* biofilms by preferential binding to α-sheet structure of PSMα1 (Bleem et al. 2017). Additionally, D-peptides, originally designed to inhibit oligomers of amyloid-β in Alzheimer’s disease, target CsgA spine segments to inhibit CsgA fibrillation and finally reduce *Salmonella typhimurium* biofilm (Perov et al. 2019). In conclusion, targeting amyloid-forming proteins to inhibit fibrillation would be helpful in addressing biofilm-related diseases (Perov et al. 2019).

A paradigm of amyloid fibrils in Gram-positive organisms is emerging in researches for *Streptococcus mutans*, where amyloid fibrils are biofilm-related issues (Besingi et al. 2017). *S. mutans* is well-known as an established cariogenic agent, dwelling on tooth surface within biofilms to cause dental caries (Lemos et al. 2019). Amyloid fibrils are visualized ubiquitously in both clinical and laboratory strains of *S. mutans* (Oli et al. 2012). Of note, P1, also named Antigen I/II, SpaP, AgB or PAc, is initially identified as a representing amyloid-forming protein in *S. mutans*, where C123 (C-terminal segment) of P1 protein is liberated separately and associates with cell-wall anchored P1 in a non-covalently way to form functional supramolecular structure regarded as amyloid fibrils (Brady et al. 2010; Larson et al. 2011; Oli et al. 2012; Heim et al. 2014, 2015; Besingi et al. 2017). More recently, biological interactions of C3 with A3VP1 (a globular region in P1) unravel that C3 fragment serves as foundation during C123-P1 interactions (Rivière et al. 2020). The interactions between C123 and P1 also likely contribute to biofilm-related events such as formation of amyloid fibrils by C123 (Rivière et al. 2020), suggesting that C3 would serve as a promising anti-amyloid target.

Managing dental caries with amyloid inhibitors, such as α-sheet peptides, are reported to be promising (Paranjapye and Daggett 2018). Apart from peptides as anti-amyloid agents, small molecules are also a rich source to need great demand for therapeutics that disperse *S. mutans* biofilms through non-microbicidal pathways (Scharnow et al. 2019). Small molecules are convenient for manufacture and can target proteins specifically that other drugs are unable to access, contributing to better curative effects with low adverse effects as well as drug resistance (Chen et al. 2019b). Various approaches have been proposed to select active molecules based on specific targets, and one reliable solution is to find new indications for existing drugs, which is a costless and fast way with a comprehensive drug library (Chong and Sullivan 2007). AA-861, targeting amyloid fibrils to inhibit both *S. mutans* and *B. subtilis* biofilms, has been screened successfully from a collection containing 480 small molecules, suggesting that screening small molecules is a novel and feasible strategy to manage biofilm-related events (Romero et al. 2013; Besingi et al. 2017).

Nevertheless, there are few researches about finding small molecules targeting specific amyloid-forming proteins and evaluating their effects on *S. mutans* biofilms. Here, we hypothesize that C3 segment can serve as a specific target for screening small molecules to impede the formation of amyloid fibrils and *S. mutans* biofilms. Inspired by rapid development of computational technology, we firstly screened for the most potent small molecule and evaluated its antimicrobial effects as well as found its potential mechanism. Based on our results, we could conclude that small molecule was applicable in reducing *S. mutans* biofilm through anti-amyloid mechanism, which also proved the effectiveness of amyloid fibrils as targets and will finally benefit in discovering new drugs for prevention and treatment of dental caries.

## Materials and methods

### In silico structure-based virtual screening

MOE (Molecular operating environment) software was utilized for in silico structure-based virtual screening. The crystal structure of C123 was obtained from Protein Data Bank (PDB, accession number 3QE5) (Burley et al. 2021), and C3 segment in C123 were used to serve as the target protein for screening. Binding energy of C3 segment and small molecules which were collected from Specs database (http://www.specs.net) was calculated within the predicted binding pocket to find promising small molecules with drug-like properties based on Lipinski’s rules (Detailed procedure was posted in Materials and Methods part in Additional file 1). Top 99 small molecules with the highest binding energy were selected, among which 94 small molecules were purchased from Specs Company (Delft, Netherlands). Fifty-five small molecules which could dissolved in DMSO at 10 mg/mL were named D1–D55 and were enrolled in further experiments. For all assays, the final concentration of DMSO was set at 1%, and wells with 1% DMSO were set as the control group.

### Bacterial strains and culture conditions

Bacterial strains, including *S. mutans* UA159 (ATCC 700610), *Streptococcus sanguinis* (ATCC 10556), *Streptococcus gordonii* (DL-1) were grown statically in BHI broth (brain heart infusion, Difco, USA) at 37 ℃ under anaerobic conditions (10% H_2_, 5% CO_2_, 85% N_2_), and adjusted to 1 × 10^6^ CFU/mL through all the experiments.

### Biofilm formation assay

Biomass of *S. mutans* biofilms was calculated through crystal violet staining for preliminary screening as described before with some modifications (Zhang et al. 2019). Noticeably, BHIs (brain heart infusion with 1% sucrose) was applied to trigger formation of biofilms as reported previously where amyloid inhibitors are still effective when *S. mutans* forms biofilm in sucrose-rich medium (Besingi et al. 2017). Briefly. *S. mutans* were grown statistically and anaerobically in BHIs broth containing small molecules from 6.25 to 100 μg/mL to a total of 200 μL in 96-well plates. After 24 h, biofilms were washed 3 times with phosphate-buffered saline (PBS) to remove unbound bacteria and fixed with absolute methanol for at least 15 min. Fixed biofilms were then stained with 0.1% crystal violet solution for 15 min, followed by gentle washing with flowing water until no existing dye appeared in blank wells. Once no visible water was detected in wells, 200 μL of 95% ethanol was added and plates were shaken vigorously for 30 min to solubilize biofilms. Finally, solubilization was transferred to new 96-well plates to denote the absorbance at 595 nm. Three independent experiments were conducted to get final biomass. Most potent small D20 and D25 were selected from preliminary screening and enrolled in future investigations. The effects of D20 and D25 on *S. mutans, S. gordonii and S. sanguinis* biofilms from 1.56 to 25 μg/mL were measured by crystal violet staining in the same manner as described above.

### CFU counts

The bactericidal effects of D20 and D25 at the concentration of 1.56–25 μg/mL were evaluated using CFU counts as described previously (Chen et al. 2021). *S. mutans*, *S. gordonii* and *S. sanguinis* cells were inoculated into BHI broth with or without small molecules in 1.5 mL Eppendorf tubes. After 24 h, the solution was serially diluted ten-fold to 10^6^ fold with PBS, and 10 μL was pipetted and placed on BHI agar plate at 37 ℃ anaerobically for 36 h (Chen et al. 2021). CFU counts were determined with three independent experiments.

### Cytotoxicity assay of HGE cells (human gingival epithelial cells)

Cell viability was evaluated with a cell counting kit-8 (Chen et al. 2021). HGE cells were firstly seeded in 96-well plates (3000 cell/100 μL) for 24 h, and then treated with medium containing 1% DMSO or medium with D20 or D25 (1.56–25 μg/mL). After incubation for another 24, 48 and 72 h, cells were washed with PBS and fresh medium (100 μL) with CCK-8 (10 μL) was added. The plates were incubated for another 1 h and were measured at OD_450_, and the cell viability was finally calculated with three independent experiments according to the manufacturer’s instructions (DojinDo, Japan).

### Biofilm imaging

*S. mutans* cells were seeded on sterile glass coverslips for 24 h to form biofilms with or without the addition of D25 at a final concentration of 6.25 μg/mL. The biofilms were then visualized by different microscopies. Confocal laser scanning microscopy (CLSM) was performed to record the distribution of live/dead bacteria and 3D architecture within biofilms. *S. mutans* biofilms were incubated on glass coverslips in 48-well plates and then rinsed with ddH_2_O to remove free-floating cells. Biofilms were then stained with 2.5 μM SYTO 9 and propidium iodide (Invitrogen, USA) as described previously (Klein et al. 2009; Ren et al. 2016; Chen et al. 2021), and imaged using CLSM with a 63 ×  lens objective (Zeiss, Germany). The three-dimensional structure was documented and quantified using COMSTAT image-processing software (Heydorn et al. 2000; Klein et al. 2009; Ren et al. 2016). For scanning electron microscopy (SEM) studies, biofilms were formed as shown in CLSM assays. Differently, biofilms were fixed with 2.5% glutaraldehyde for at least 3 h, dehydrated using graded ethanol (30–100%), treated with tert-butanol for three times and afterwards, freeze-dried and sputtered with gold. Images were recorded using SEM system (FEI, QUANTA2000, Czech Republic). Atomic force microscopy (AFM) was utilized to portray the morphology of single bacterial cells in biofilms as described previously (Sharma et al. 2014). After incubation on sterile coverslips in 12-well plates for 24 h, the medium was decanted and the biofilms were immersed in PBS for observation by AFM with tapping-mode (Bruker, USA).

### Quantification analysis and TEM images of bacterial amyloid fibrils

*S. mutans* cells were grown in BHI broth with 1% DMSO or BHI broth with 6.25 μg/mL D25 to quantify amyloid fibrils with ThT (Thioflavin T) fluorescence assays. ThT could bind to β-sheet structures in amyloid fibrils specifically and ThT assay is regarded as the gold standard to explore the formation of amyloid fibrils, where the intensity of ThT could reflect the quantification of amyloid fibrils (LeVine 1999; Bleem et al. 2017; Paranjapye and Daggett 2018). Free-floating bacteria were incubated anaerobically at 37 ℃ in 1.5 mL Eppendorf tubes for 24 h. Then the bacterial cells were harvested by centrifugation (12,000*g*, 10 min, 4 ℃) and resuspended with PBS. PBS with 1% DMSO was set as the blank control. Samples were mixed with 40 μM ThT in equal volume and the mixture was pipetted into opaque 96-well plates. Plates were incubated for 15 min, and fluorescence intensity was measured at 438 nm excitation and 495 nm emission (Romero et al. 2010; Schwartz et al. 2012, 2016). Fluorescence intensity was modified by subtracting intensity of blank control. Additionally, samples were imaged via TEM to depict the structure of amyloid fibrils as stated previously (Schwartz et al. 2012). Briefly, samples were absorbed onto formvar-coated copper grides, stained with 3% phosphotungstic acid for 2 min and washed with sterile ddH_2_O. TEM images were recorded through Hitachi TEM system. Moreover, ThT fluorescence was also measured in *S. mutans* biofilms with some modifications (Chen et al. 2019a). *S. mutans* biofilms were firstly incubated in 6-well plates for 24 h in same conditions as described above. Then samples were scrapped, resuspended with PBS and mixed with ThT to obtain fluorescence intensity. Simultaneously, samples were prepared for TEM images to depict morphology of amyloid fibrils in *S. mutans* biofilms.

### Fibrilization of purified C123 in vitro

ThT assays were also applied to explore the effects of D25 on amyloid-forming protein C123. At the very beginning, we got access to purified C123 with the same protocols as reported previously (Chen et al. 2019a). Protein was diluted with PBS to 0.1 mg/mL and mixed with PBS with 1% DMSO or PBS with D25 at 6.25 μg/mL, then the mixture in 1.5 mL Eppendorf tubes was stirred at 4 ℃ for 72 h (Besingi et al. 2017). ThT fluorescence were measured in the presence or absence of purified protein, and final ThT fluorescence were calculated by subtracting ThT fluorescence without protein. Moreover, samples were also prepared for TEM imaging as described above.

### Gene expression

Quantitative reverse transcriptase PCR (qRT-PCR) was used to evaluate the effects of D25 on the expression levels of amyloid-related genes. Biofilms were grown with BHIs broth in 6-well plates in the presence or absence of D25 at 6.25 μg/mL. After 24 h, biofilms were scraped, centrifugated (12,000*g*, 10 min, 4 ℃) and harvested into 1.5 mL Eppendorf tubes. The bacterial cells were lysed with 500 μL lysis buffer (20 mg/mL lysozyme; 20 Mm Tris–HCl, PH  = 8; 2 mM EDTA; 1.2% Triton) for at least 2 h and treated with proteinase K for at least 30 min. RNA was extracted from lysate using manufacturer’ protocols. The purification of the lysate was determined using Nanodrop 2000 (Thermo Fisher, USA) followed by reverse-transcription of interest RNA. Comparative expression of genes were ultimately quantified according to the 2^−ΔΔCT^ method (Detailed procedure was posted in Materials and Methods part in Additional file 1: Table S1).

### Statistical analysis

Data are mean  ±  standard deviations from three independent experiments. Statistical analysis of all data were performed with GraphPad Prism 7 (GraphPad Software, San Diego, USA) using analysis of variance (ANOVA) and Student’s two-sample t test. Dunnett’s multiple comparison were used to compare differences between control group and treated group. Significant differences were set at the 95% confident level.

## Results

### Preliminary structure-based virtual screening for finding potent small molecules

**Fig. 1:**
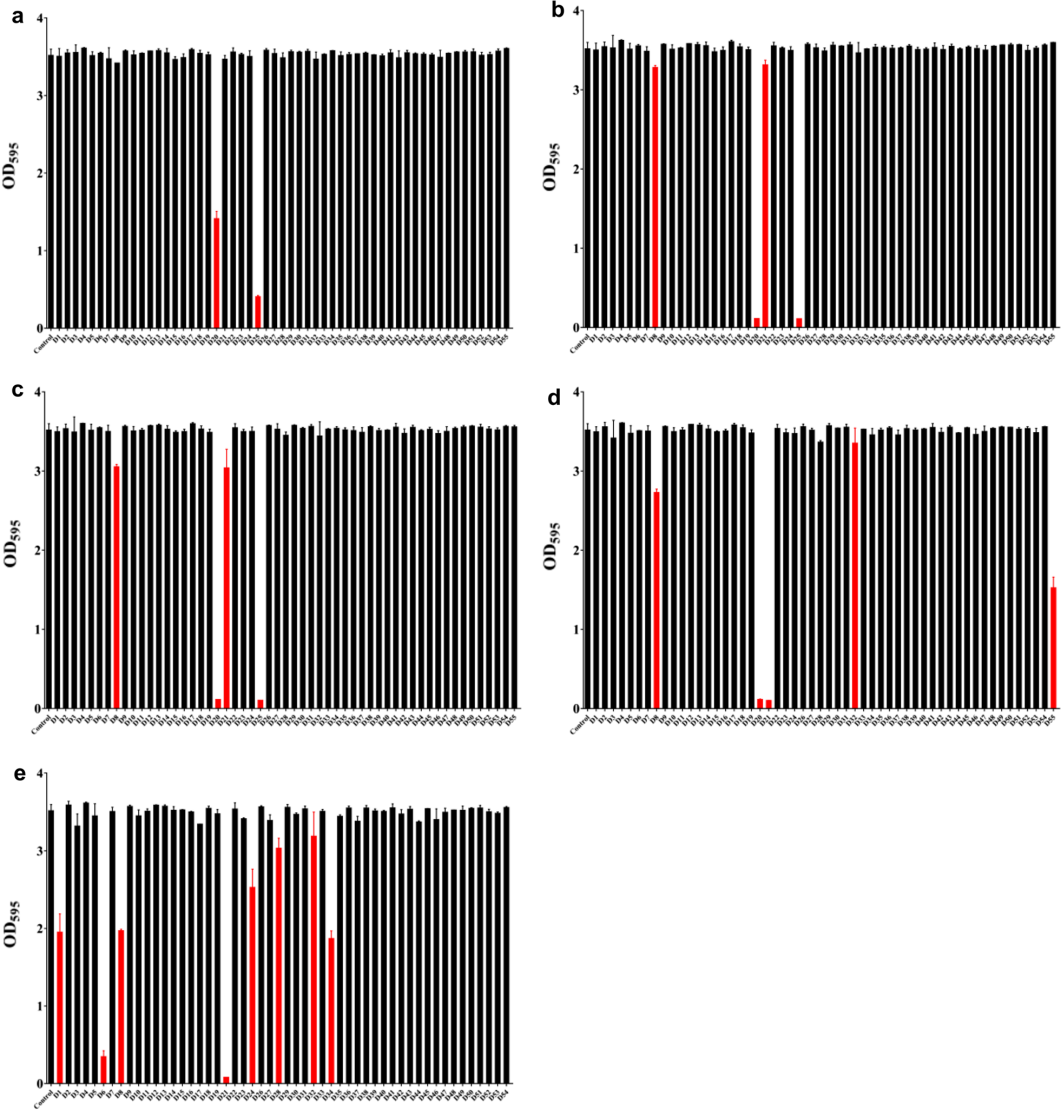
Effects of compounds at **a** 6.25 μg/mL, **b** 12.5 μg/mL, **c** 25 μg/mL, **d** 50 μg/mL and **e** 100 μg/mL on *S. mutans* biofilm were evaluated in vitro by crystal violet staining. Data are mean  ±  standard deviations from three independent experiments. The red bars denoted that differences of biomass in control and these groups were statistically significant (*P*  < 0.05)

Based on Specs database, we conducted structure-based virtual screening method to find small molecules with higher docking scores with C3. The crystal structure of C3 in C123 were obtained from Protein Data Bank and utilized in the screening. Totally, we screened about 2,20,000 small molecules and listed top 99 small molecules (Additional file 1: Figure S1). We purchased 94 small molecules with enough mass from Specs company, and 55 small molecules were dissolved in DMSO to get stocking solution, which were then diluted into gradient concentrations and finally enrolled in the crystal staining assay (Additional file 1: Table S2; Fig. 1). Notably, initial concentrations were set for D20, D55 at 50 μg/mL and D25 at 25 μg/mL respectively, owing to their solubility when added into different mediums.

According to our results, small molecules D1, D6, D8, D21, D24, D28, D32, D34 and D55 showed inhibitory effects against *S. mutans* biofilms at higher concentrations (more than 6.25 μg/mL). At lower concentrations (6.25 μg/mL), only D20 and D25 corroborative showed inhibitory effects on biofilms (Fig. 1). Thus, we recruited D20 and D25 for detailed investigations.

### Better performance of D25 on *S. mutans* biofilms, free-floating bacteria and cytotoxicity

**Fig. 2:**
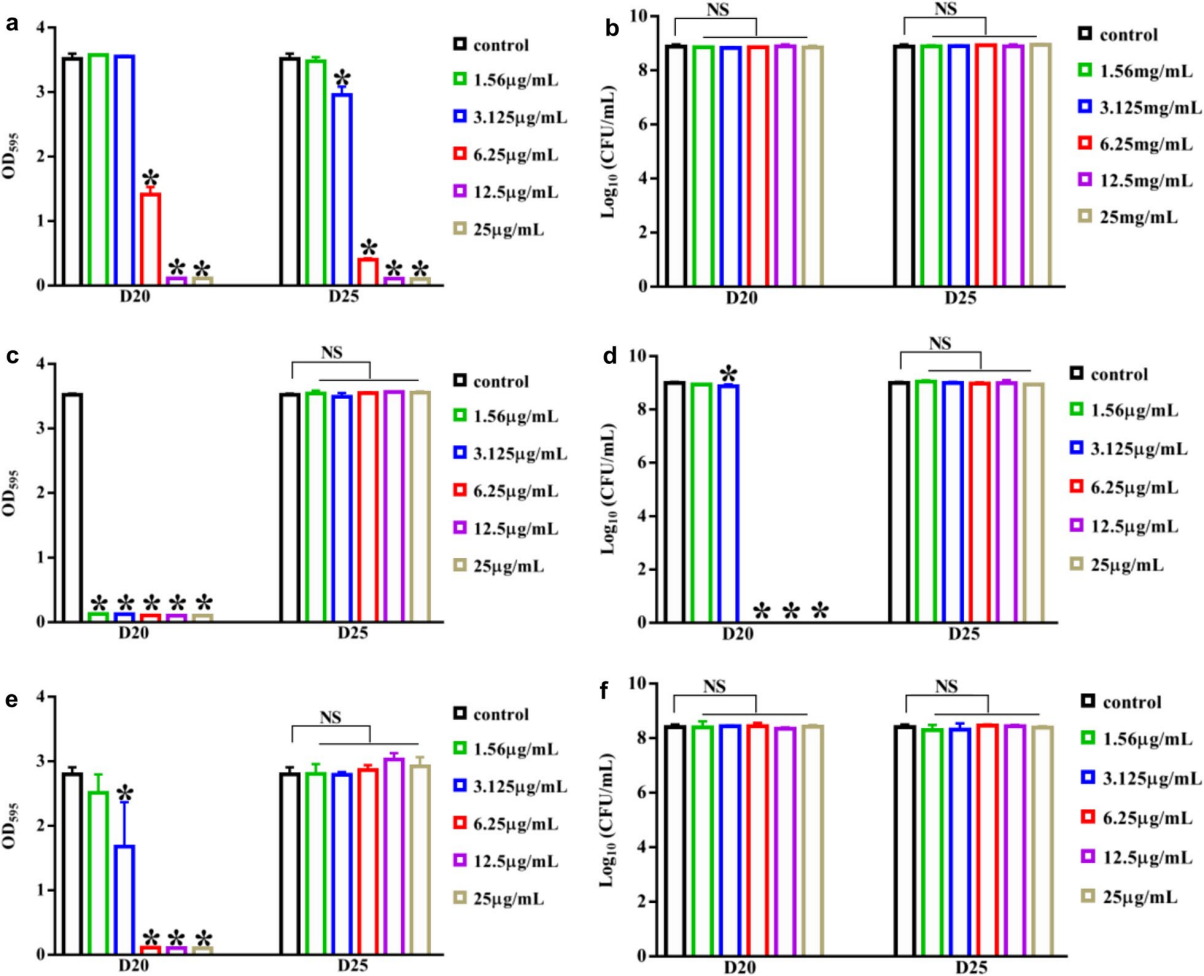
Effects of D20, D25 on *S. mutans*, *S. gordonii, S. sanguinis* biofilms as well as their free-floating bacterial cells. All experiments were conducted at the concentration of 1.56–25 μg/mL. Effects on *S. mutans*, *S. gordonii*, *S. sanguinis* biofilm was evaluated by crystal violet staining. **a** *S. mutans* biofilm, **c** *S. gordonii* biofilm, **e** *S. sanguinis* biofilm. Data are mean  ±  standard deviations from three independent experiments. Effects of D20 and D25 on free-floating bacterial cells were measured by means of CFU counts. **b** *S. mutans*, **d** *S. gordonii*, **f** *S. sanguinis*. Data were mean  ±  standard deviations from three independent experiments. *Differences were statistically significant when compared with control group (*P*  < 0.05). *NS* differences were not statistically significant when compared with control group

In light of strong antibiofilm effects of D20 and D25 at the concentration of 6.25 μg/mL, we wondered whether they would function at lower concentrations. We then evaluated D20 and D25 for antibiofilm and bactericidal effects on *S. mutans*, *S. gordonii* and *S. sanguinis* as well as cytotoxicity at the concentration of 1.56–25 μg/mL. Effects of D20 and D25 on *S. mutans*, *S. gordonii* and *S. sanguinis* biofilms from 1.56 to 25 μg/mL were demonstrated by crystal violet assay (Fig. 2). It was obvious that D25 had inhibitory impacts on *S. mutans* biofilms at the concentration of 3.125–25 μg/mL, in addition, at 6.25–25 μg/mL, D25 inhibited more than 50% *S. mutans* biofilms. Nevertheless, biofilms formed by *S. gordonii* and *S. sanguinis* were not impaired in the presence of D25. For D20, it inhibited *S. mutans* biofilms at the concentration of 6.25–25 μg/mL. Unlike D25, D20 tended to show more inhibitory effects against *S. gordonii* biofilms from 1.56 to 25 μg/mL and *S. sanguinis* biofilms from 3.125 to 25 μg/mL respectively. Furthermore, for their effects on free-floating cells, D25 did not impacted *S. mutans*, *S. gordonii* and *S. sanguinis* cells at the concentration of 1.56–25 μg/mL. On the contrary, D20 presented selective bactericidal effects against *S. gordonii* cells at the concentration of 3.125–25 μg/mL, but did not show bactericidal effects on other two streptococci (Table 1; Fig. 2).

**Table 1:** Minimum concentration of tested small molecules (μg/mL)

Bacteria	Biofilms		Free-floating cells	
	D20	D25	D20	D25
*S. mutans*	6.25	3.125	–	–
*S. gordonii*	1.56	–	3.125	–
*S. sanguinis*	3.125	–	–	–

**Fig. 3:**
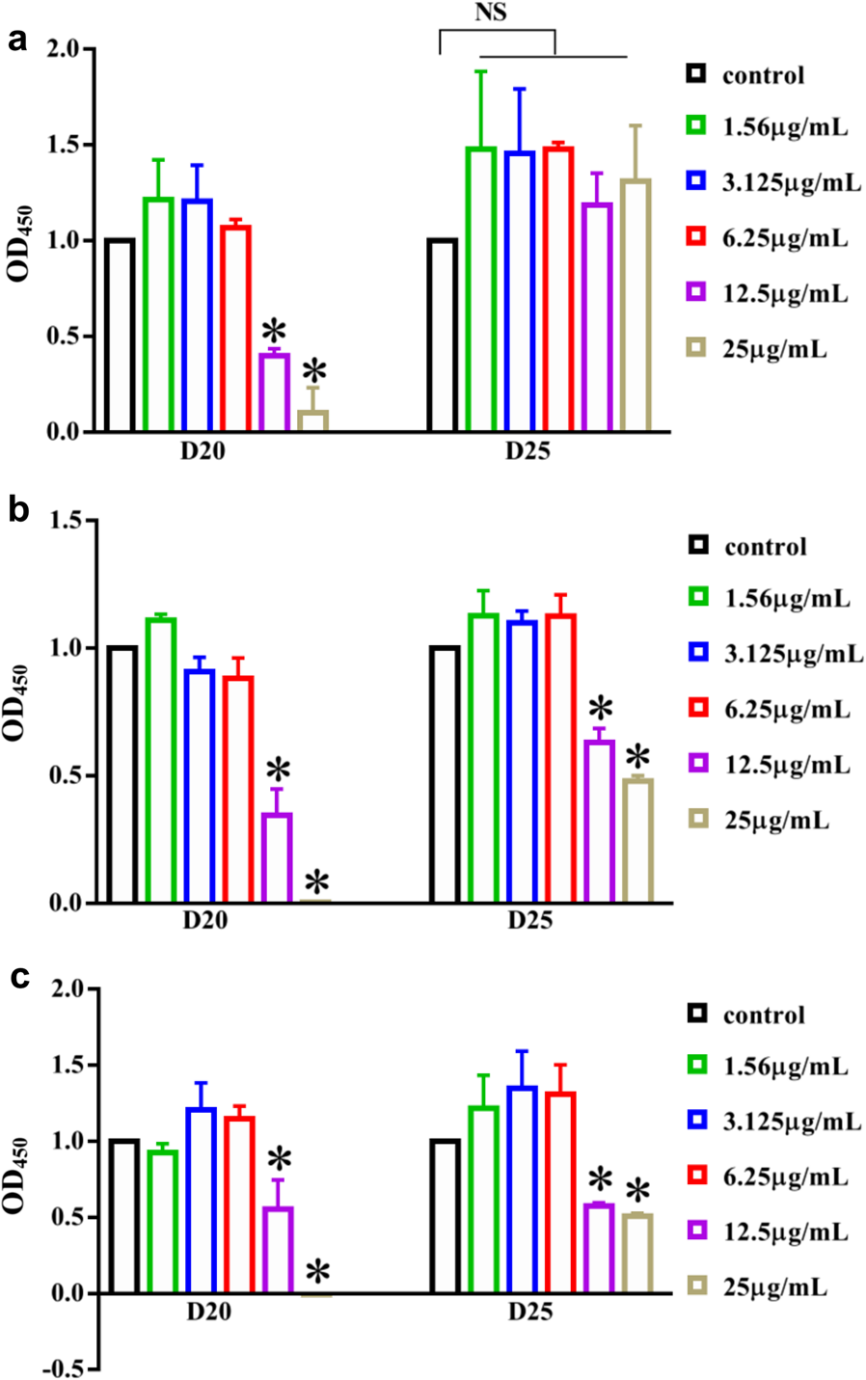
Cytotoxicity of D20 and D25 on HGE cells. **a** 24 h, **b** 48 h and **c** 72 h. Data were mean  ±  standard deviations from three independent experiments. *Differences were statistically significant when compared with control group (*P*  < 0.05). *NS* differences were not statistically significant when compared with control group

**Fig. 4:**
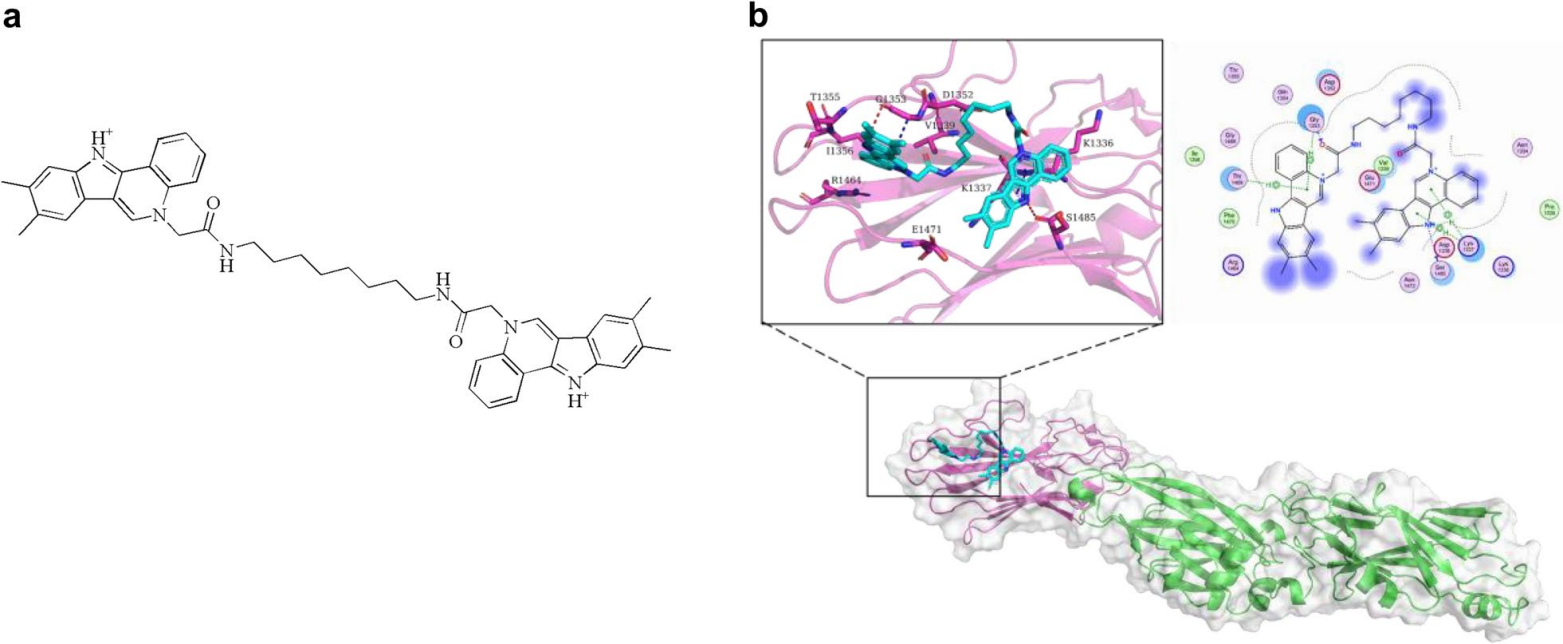
**a** The chemical structure of D25. **b** The binding mode and binding site of D25 with C3

Cytotoxicity is also an important property when considered future application. Cell viability was documented on HGE cells for 24–72 h (Fig. 3). D25 did not showed inhibitory effects on cell proliferation at tested concentration for 24 h (*P*  > 0.05), but inhibited cell viability at 12.5–25 μg/mL for 48 h and 72 h (*P*  < 0.05). While D20 inhibited the growth of HGE cells at 12.5–25 μg/mL from 24 to 72 h (*P*  < 0.05). In all, we concluded that D25 showed selectively inhibitory capacity on *S. mutans* biofilms when compared with other two streptococci. Simultaneously, D25 did not influence the growth of free-floating bacterial cells and HGE cells at 1.56–6.25 μg/mL. Combined with the inhibitory efficacy of D25 on biofilms, we recruited D25 at the concentration of 6.25 μg/mL for detailed experiments. The chemical structure of D25 and its predicted binding mode with C3 were shown in (Fig. 4).

### Morphological changes of *S. mutans* biofilms

**Fig. 5:**
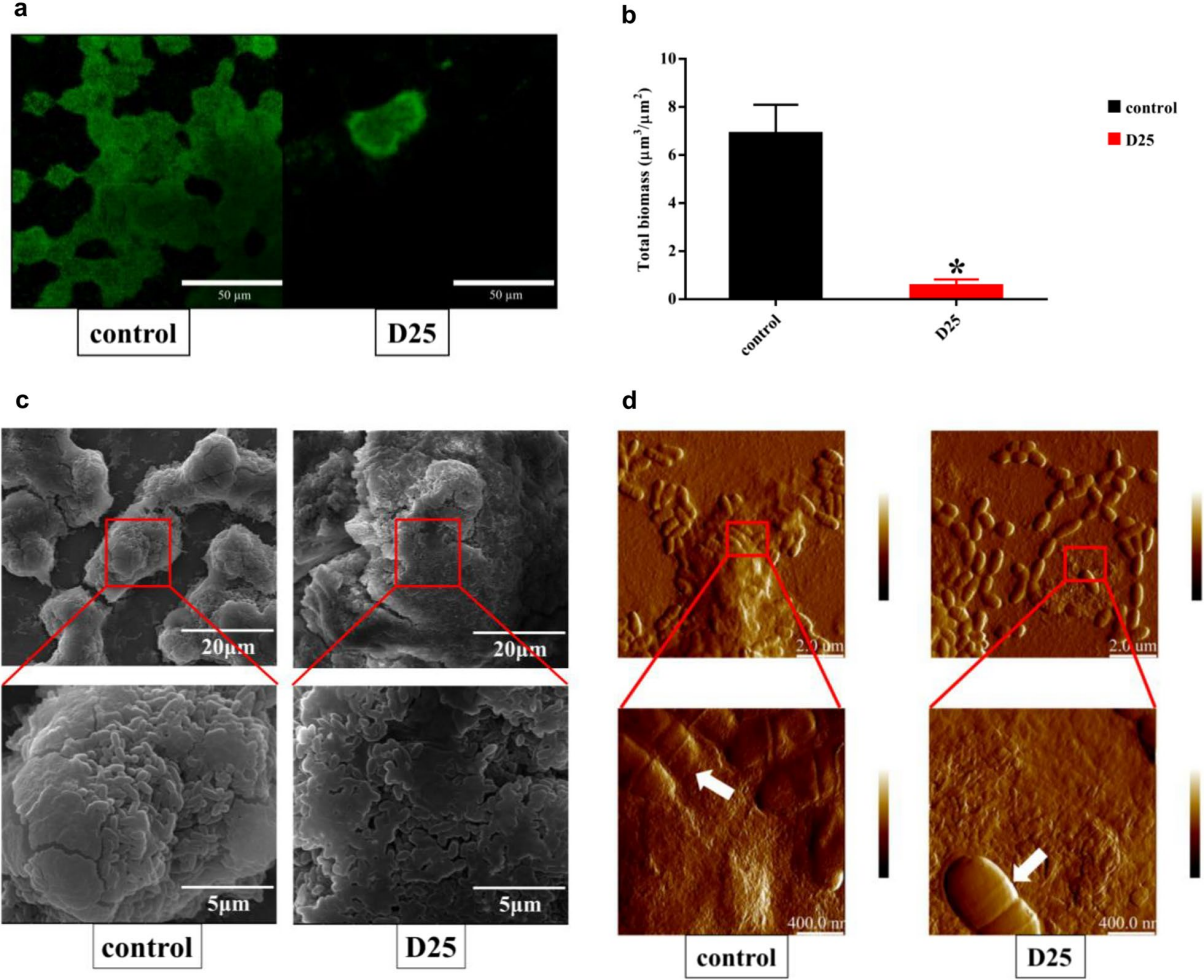
Morphological changes of *S. mutans* biofilms when treated with or without D25 at the concentration of 6.25 μg/mL. **a** Confocal images (merged images of dead/live bacteria) showed the inhibitory effects of D25 on *S. mutans* biofilms (Green, live bacteria, SYTO9; red, dead bacteria, PI). Images were taken at 63 ×  magnification. **b** Quantification analysis of biomass were evaluated with Comstat 2. *Differences were statistically significant when compared with control group (*P*  < 0.05). **c** Architecture of 24-h *S. mutans* biofilms was documented by scanning electron microscopy. Images were acquired at the magnification of 5000 ×  and 20,000 × . **d** The morphological changes of single bacterial cell in biofilms were recorded via atomic force microscopy. White arrows represented *S. mutans* cells

It was an efficient way to utilize electronic microscopies for sake of recording visual changes. In this study, we applied confocal laser scanning microscopy (CLSM), scanning electron microscopy (SEM) and atomic force microscopy (AFM) to portray morphological changes of *S. mutans* biofilms. Firstly, we applied CLSM to record the biofilm in the presence or absence of D25 (Fig. 5a). It was evident that the biofilm was impeded when treated with D25. Simultaneously, the biomass in D25 group was markedly decreased than in control group (*P*  < 0.05) (Fig. 5b). Next, SEM was used to recorded inner changes of structure in *S. mutans* biofilms. There was no obvious changes in the shape and size of bacterial cells in D25 group. In addition, all the bacterial cells linked and connected closely to form complex structure (Fig. 5c). Furthermore, the morphology of single bacterial cell in biofilms was recorded via atomic force microscopy (AFM) (Fig. 5d). The shape of the bacterial cells in the treated group was identical to it in control group, which corresponded to the nonfatal properties of D25 as described above.

### Antibiofilm activities via amyloid-dependent mechanism

**Fig. 6:**
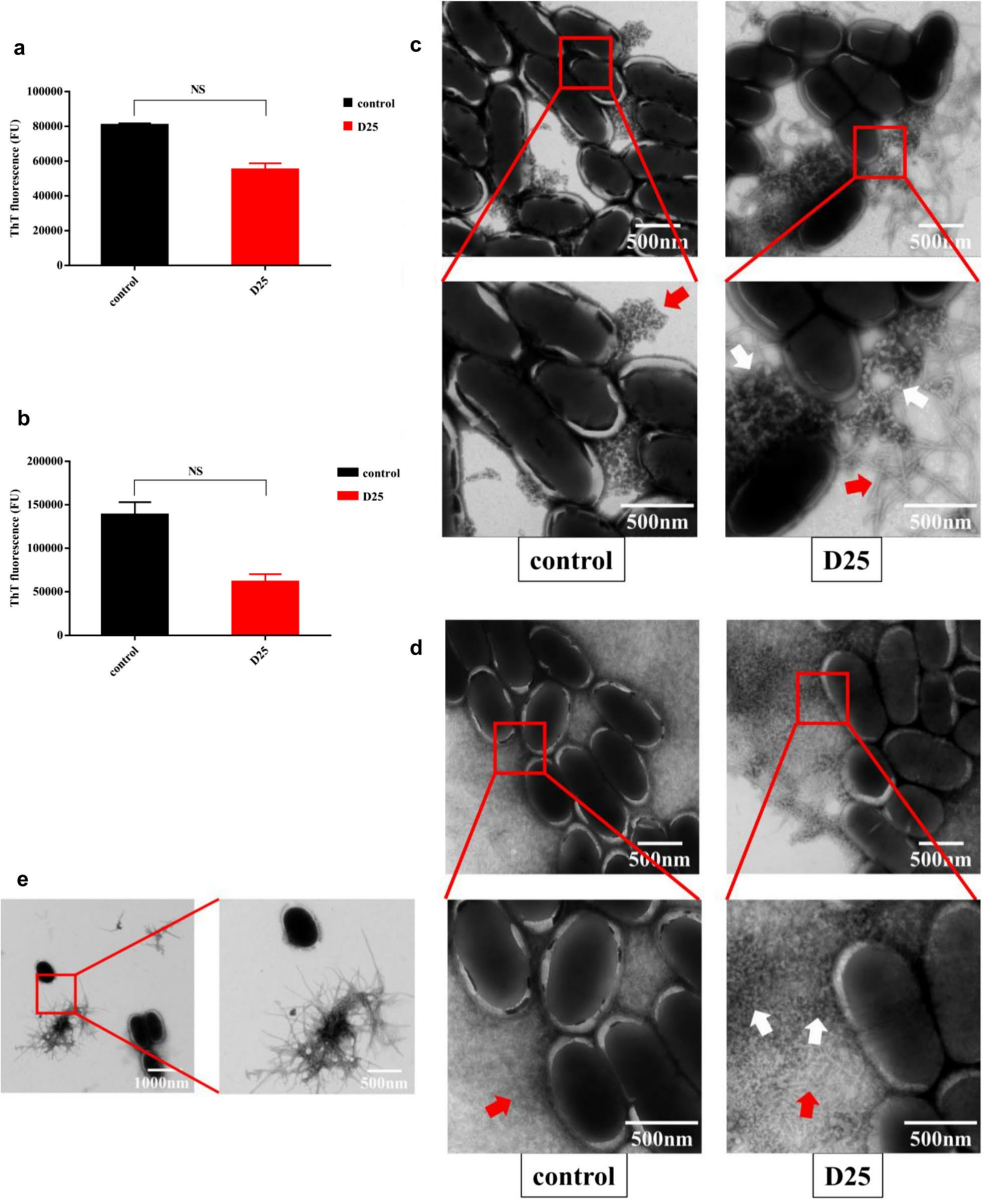
Effects of D25 on bacterial amyloid fibrils. Fluorescence intensity of free-floating bacterial cells and biofilms were utilized to document effects of D25 on amyloid fibrils. **a** free-floating bacterial cells. **b** biofilms. Data were presented as mean  ±  standard deviations from three independent experiments. *NS* differences were not statistically significant when compared with control group (*P*  < 0.05). TEM (transmission electron microscopy) images of amyloid fibrils in **c** free-floating bacterial cells and **d** biofilms were recorded in the presence or absence of D25. White arrows represented amorphous aggregates and red arrows represented amyloid fibrils. **e** Free amyloid fibrils and single bacterial cell without obvious amyloid fibrils around were visualized in biofilms by TEM images

To elucidate the mechanism of D25 for their antibiofilm ability, we quantified amyloid fibrils by means of ThT assays, and depicted their morphological changes through transmission scanning microscopy (TEM), ultimately we evaluated the transcription level of several related genes to describe the mechanism more detailly. At the very beginning, the effects of D25 on amyloid fibrils in free-floating bacterial cells and in biofilms were investigated via ThT assays quantitatively (Fig. 6a, b). According to our data, we found that ThT uptake of amyloid fibrils in free-floating cells and in biofilms were reduced in the presence of D25, but the differences were not statistically significant compared with control group (*P * > 0.05).

Moreover, considering the importance of confirming morphological changes in amyloid fibrils, TEM was utilized to identify the structure with D25 added (Fig. 6c). Generally, in the absence of antibiofilm agents, amyloid fibrils would be atypical with few amount in the free-floating bacterial cells as described previously (Chen et al. 2019a). Interestingly, in response to D25, morphological changes were obvious due to the occurrence of amorphous aggregates especially in free-floating bacterial cells. These amorphous aggregates were not definitive structures of amyloid fibrils and could not be found in the absence of D25. Amorphous aggregates arranged irregularly to separate amyloid fibrils from cell surface. Furthermore, fibrous structures around amorphous aggregates were long, scattered and apparent (Fig. 6c), differed from typical amyloid fibrils in biofilms (Fig. 6d). In biofilms, bacterial cells were surrounded by dense and numerous amyloid fibrils as described previously (Chen et al. 2019a). In addition, when treated with D25, only a small number of amorphous clusters were detected around the cell surface (Fig. 6d), and they were harder to find than in free-floating bacterial cells. However, this did not indicate the reduction of amorphous aggregates in *S. mutans* biofilms, but it was likely that amorphous aggregates were shed underneath ascribe to the large number of amyloid fibrils. Besides, it was unique that in some regions, fibrillar structure were liberated away from cell surface, along with single and disperse bacteria without amyloid fibrils (Fig. 6e), which could not be detected in control group. Thus, to some extent, in the presence of D25, morphological changes in amyloid fibrils were obvious and meaningful for us to clarify the underneath mechanism.

**Fig. 7:**
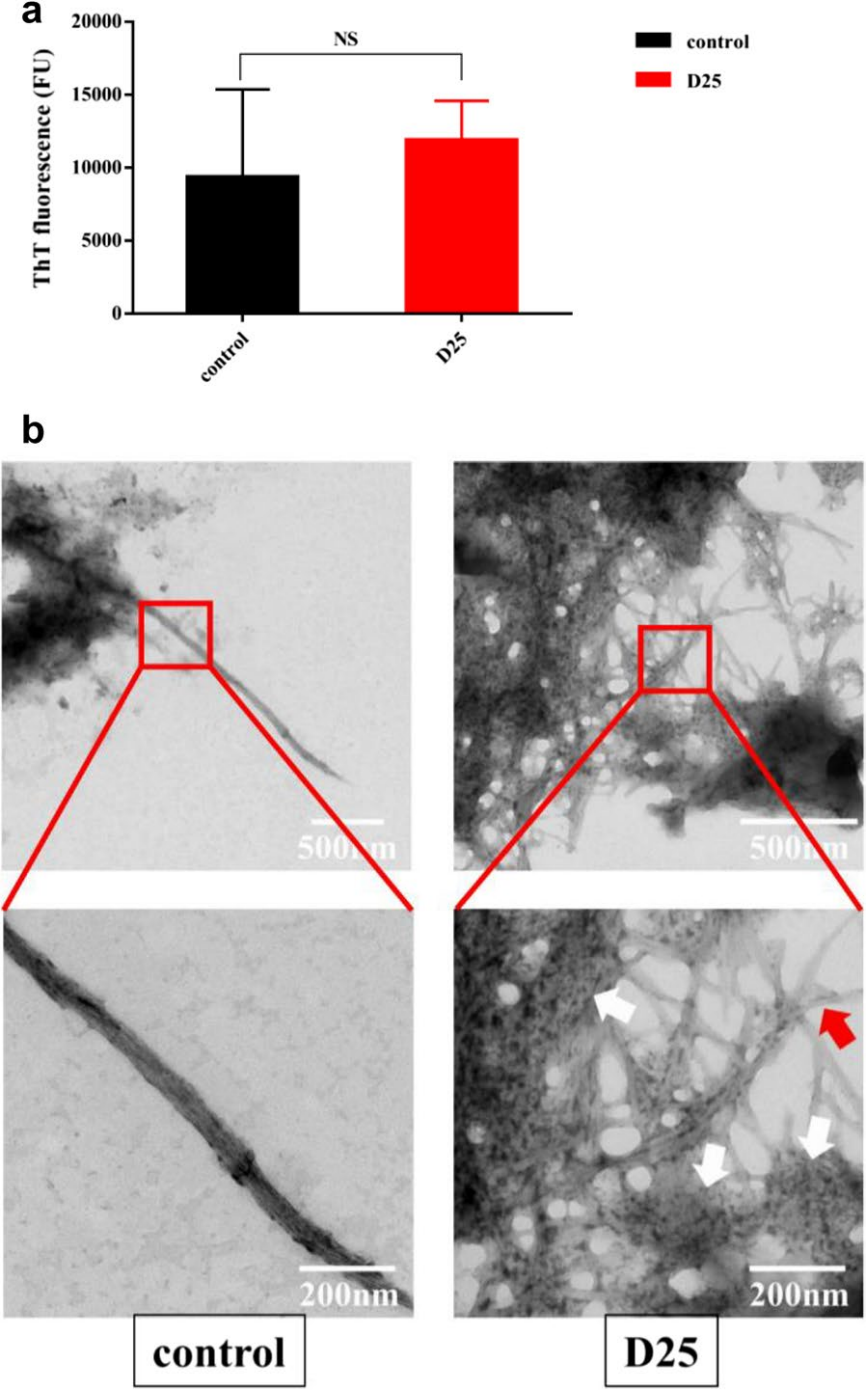
Effects of D25 on purified proteins. **a** Fluorescence intensity of purified C123 were tested via ThT assays. Values were obtained from three independent experiments and shown as mean  ±  standard deviations. *NS* differences were not statistically significant when compared with control group (*P*  < 0.05). **b** TEM images of amorphous aggregates formed by C123 in vitro. Typical fibrous structures could be found in control group. Only in D25 group, amorphous aggregates were obvious with long stem amyloid fibrils. White arrows represented amorphous aggregates and red arrows represented amyloid fibrils

To verify the effects of D25 on C123 fragments for their ability to form amyloid fibrils, we synthesized C123 fragments in vitro as mentioned before (Chen et al. 2019a). Purified C123 fragments were mixed with or without small molecules and stirred at 4 ℃ for 3 days. Interestingly, there was no reduction in ThT fluorescence with D25 treatment (*P*  > 0.05) (Fig. 7a). For TEM images, we found typical amyloid fibrils (about 55 nm) in control groups (Fig. 7b) as reported previously (Besingi et al. 2017; Chen et al. 2019a). Notably, amorphous aggregates were obvious in D25 treated group, surrounded by dense amyloid fibrils (Fig. 7b). Hereinto, amorphous aggregates ranged in shape and size in different regions. Coupled amyloid fibrils were long but less wider (about 24 nm) compared with typical amyloid fibrils, and interestingly, they were extended from amorphous aggregates at different directions.

**Fig. 8:**
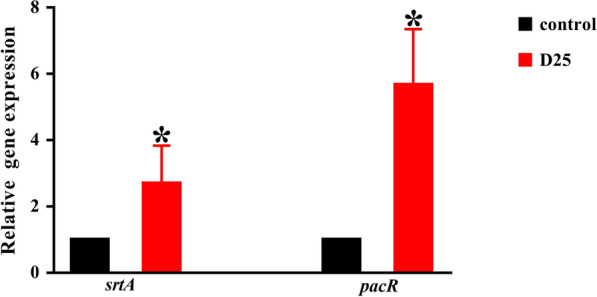
Effects of D25 on the expression of P1 related genes (*srtA* and *pacR* genes). The results from real-time reverse transcription-quantitative PCR were calculated by the 2^−ΔΔCT^ method. All results were shown as mean  ±  standard deviations from three independent experiments. *Differences were statistically significant when compared with control group (*P*  < 0.05)

Finally, quantitative reverse transcriptase PCR (qRT-PCR) was applied to quantify the expression levels of P1 related *srtA* and *pacR* genes with D25 added (Fig. 8). The presence of D25 upregulated the transcription level of *srtA* gene by about 2.7-fold (*P*  < 0.05). Simultaneously, the expression level of *pacR* gene was upregulated by about 5.7-fold (*P*  < 0.05).

## Discussion

Due to the vital role of *S. mutans* biofilm formation in dental caries, targeting biofilm-related factors will facilitate discovery and application of new anticaries compounds (Cegelski et al. 2009; Oli et al. 2012). It has been proposed that *S. mutans* biofilms would be inhibited with several amyloid inhibitors (Oli et al. 2012), suggesting a novel antibiofilm strategy in amyloid-dependent way. Thus, in this study, we aimed to find potential inhibitors targeting amyloid-forming proteins with the capacity to reduce *S. mutans* biofilm formation and ultimately benefit in the prevention and treatment of dental caries.

In light of our results, we could conclude that the selected small molecule D25 was reliable and promising as antibiofilm agents. Unlike the broad antibiofilm effects of D20 on *S. mutans*, *S. gordonii* and *S. sanguinis*, which would function by means of bactericidal effects or in other pathways without destroying bacterial cells, the selectivity of D25 would be an advantage for its future application, as D25 did not affect the growth and biofilm formation of *S. gordonii* and *S. sanguinis*, and this may account for the specific and unique sequence of C3 domain used in screening. Virtual screening, which is an effective computational methodology aided in the discovery of new drugs, were utilized in this study (Baig et al. 2016; Ghislat et al. 2021; Kimber et al. 2021; Zorn et al. 2021). Generally, structural information of protein is the core of computational virtual screening that variations in crystal structures would result in different binding pockets and binding affinity (Baig et al. 2016; Ochoa et al. 2017; Rivera-Pérez et al. 2019). The crystal structure of C123 segment is highly conservative and nearly identical to all *S. mutans* strains, but heterogeneous compared with other oral streptococci in structural superpositions, which correspond to unique electrostatic surfaces and recognition properties, and subtle changes appeared in structural properties would ultimately result in the distinct characteristics of binding (Larson et al. 2011; Nylander et al. 2011; Esberg et al. 2012, 2017; Yang et al. 2019; Schormann et al. 2021). Therefore, by means of different binding ability, we could select small molecules with high selectivity on specific targets.

We could rationalize that D25 inhibited biofilm formation via amyloid-dependent way. We found that effects of D25 on ThT fluorescence in free-floating bacterial cells, biofilms and purified C123 were not significant compared with control group. Nevertheless, morphological changes were obvious in TEM images. It has been highlighted the importance of TEM to confirm amyloid formation owing to non-specific characteristics of dye-uptake assays (Besingi et al. 2017). Hereinto, the presence of amorphous aggregates were noticeable in response to D25. Amorphous aggregates is the other type of aberrant aggregates present in disordered process, where amyloidogenic proteins stack in a random way rather than form amyloid fibrils (Stranks et al. 2009; Yoshimura et al. 2012). In some circumstances, monomers which favor oligomerization other than fibrillization would tend to form amorphous aggregates, and conformational changes would in turn lead to a greater hydrophobic surface, promote self-tapping process and ultimately benefit in fibrillization (Necula et al. 2007; Stranks et al. 2009; Yoshimura et al. 2012; Vetri and Foderà 2015). The occurrence of mature amyloid fibrils around amorphous aggregates was a corroborative evidence showing their nucleation effects (Stranks et al. 2009; Yoshimura et al. 2012; Vetri and Foderà 2015). Therefore, we can explain that due to a feedback mechanism, amorphous aggregates ultimately lead to formation of amyloid fibrils in *S. mutans* biofilms visualized in TEM images shown in Fig. 7b (Vetri and Foderà 2015). Even though, we are still unable to demonstrate that D25 had no impacts on production of amyloid fibrils based on ThT data. The fluorescence characteristics of amorphous aggregates formed in this study were still puzzling, thus we could not eliminate their effects on ThT uptake. Further exploration is urgent and necessary to detailly evaluate physical and biological properties of amorphous aggregates. In all, we can conclude that D25 induced the formation of amorphous aggregates, which would finally interfere the formation of *S. mutans* biofilms.

In this study, C3 segment seems to be feasible as anti-amyloid target. C3 segment is foundation between C123-P1 interactions which corresponds to connections between amyloid fibrils and cell surface (Rivière et al. 2020). The interactions were found to be attenuated due to the presence of D25, which prompted formation of amorphous aggregates and liberation of amyloid fibrils. Amorphous aggregates tended to show weaker interactions with cell surface as depicted in TEM images. Moreover, dissociated fibril clusters and single bacterial cell were found in D25 group, indicating the liberation of amyloid fibrils from cell surface and dissociation of biofilms. Accordingly, these findings reinforce the role of amyloid fibrils in *S. mutans* biofilms that they would account for stability of matrix scaffold (Besingi et al. 2017). It coincides with amyloid fibrils in *B. subtilis* and *S. aureus* biofilms that have been proposed to provide structural integrity and assist in formation of biofilms (Romero et al. 2010; Taglialegna et al. 2016). To detail, we can rationalize that D25 could impact C3-A3VP1 interactions to destroy the integrity of amyloid scaffold, which would ultimately result in diminished biofilm formation.

As far as molecular mechanism was concerned, we analyzed the transcription level of several amyloid related genes quantitatively. A large number of data suggest that bacterial cells interact or respond to a variety of genes when they are influenced with external stimulus (Wen et al. 2006). Amyloid formation was firmly related to *srtA* gene and *pacR* gene. Of note, *srtA* gene mediates the activity of sortase to regulate the linkage of cell surface proteins to bacterial cell walls (Li et al. 2013). Without enough functional sortase, P1 could not anchor to cell walls and form amyloid fibrils (Oli et al. 2012; Li et al. 2013). In addition, *pacR* regulates the production of surface proteins P1, which is involved in the formation of amyloid fibrils (Oli et al. 2012). Based on our results, we could find more expression in *srtA* and *pacR* genes due to the presence of D25. Not surprisingly, the occurrence of amorphous aggregates would result in higher expression levels as the negative feedback regulation to promote production of P1 and sortase so as to form amyloid fibrils. Consequently, this suggests that D25 would affect amyloid formation in a genetic level.

There are still some limitations in our study. In terms of our results, there are no evidence of interaction sites between D25 and C123, even though by means of molecular docking, we could speculate D25 competed binding sites for C3 with A3VP1 (Rivière et al. 2020; Fig. 4). In addition, D25 would function under more than amyloid-dependent mechanism, as several virulence related genes were also influenced (Additional file 1: Figure S2). Moreover, owing to restricted application time and weak scavenging capacity for mature biofilms, which were found to have no effects on pre-formed 24-h biofilms at the concentration of 1.56–25 μg/mL and only have effects on pre-formed 4-h biofilms at the concentration of 6.25 μg/mL (Detailed procedure was posted in Materials and Methods part in Additional file 1: Figure S3), we need to modify and develop new antimicrobial drugs based on D25.

In summary, our study demonstrated that C3 could be a feasible target aided in finding potent small molecules. Small molecule D25 impaired biofilm formation by means of inducing formation of amorphous aggregates and impairing the integrity of amyloid scaffold. Hereof, targeting amyloid fibrils is a novel and promising strategy to reduce biofilms and ultimately reduce dental caries.

## Supplementary Information

**Additional file 1: Figure S1.** The flowchart of structure-based virtual screening. **Figure S2.** Effects of compound D25 on the expression of *vicR*, *brpA*, *comDE*, *atpD *and *relA *genes. The results from qRT-PCR were obtained with the 2^−ΔΔCT^ method. All results were shown as mean ± standard deviations from three independent experiments. *Differences were statistically significant when compared with control group (*P *< 0.05). **Figure S3.** Effects of D25 on biofilms at different concentrations and time intervals. **a** *S. mutans *biofilms formed after 24 h were treated with different concentrations of D25. *NS* differences were not statistically significant when compared with control group. **b** Effects of D25 on *S. mutans *biofilms formed after 4, 8, 16 and 24 h. *Differences were statistically significant when compared with control group (*P *< 0.05). *NS* differences were not statistically significant when compared with control group. **Table S1.** Primers used in this study for qRT-PCR to quantify gene expression level. **Table S2.** Top 99 small molecules selected by targeting C3 segment.

## Data Availability

The datasets generated and analysed during the current study are available from the corresponding author on reasonable request.

## Abbreviations

*S. mutans*: *Streptococcus* *mutans*
*S**.** aureus*: *Staphylococcus aureus*
*B. subtilis*: *Bacillus subtilis*
*S. gordonii*: *Streptococcus gordonii*
*S. sanguinis*: *Streptococcus sanguinis*
MOE: Molecular operating environment
BHI: Brain heart infusion
HGE cells: Human gingival epithelial cells
ThT: Thioflavin T
