# Supplementary material for: Small molecule targeting amyloid fibrils inhibits Streptococcus mutans biofilm formation
Source: AMB Express. 2021 Dec 17;11:171. doi: 10.1186/s13568-021-01333-2 (PMC8683520; doi:10.1186/s13568-021-01333-2)
Supplement: Supplementary file 1 — Additional file 1: Figure S1. The flowchart of structure-based virtual screening. Figure S2. Effects of compound D25 on the expression of vicR, brpA, comDE, atpD and relA genes. The results from qRT-PCR were obtained with the 2−ΔΔCT method. All results were shown as mean ± standard deviations from three independent experiments. *Differences were statistically significant when compared with control group (P < 0.05). Figure S3. Effects of D25 on biofilms at different concentrations and time intervals. a S. mutans biofilms formed after 24 h were treated with different concentrations of D25. NS differences were not statistically significant when compared with control group. b Effects of D25 on S. mutans biofilms formed after 4, 8, 16 and 24 h. *Differences were statistically significant when compared with control group (P < 0.05). NS differences were not statistically significant when compared with control group. Table S1. Primers used in this study for qRT-PCR to quantify gene expression level. Table S2. Top 99 small molecules selected by targeting C3 segment. [file 13568_2021_1333_MOESM1_ESM.pdf]

AMB Express

Small molecule targeting amyloid fibrils inhibits *Streptococcus mutans* biofilm formation

Yuanyuan Chen[1,3], Guxin Cui[1,3], Yuqi Cui[1,3], Dongru Chen[2,3*], Huancai Lin[1,3*]

[1] Department of Preventive Dentistry, Hospital of Stomatology, Sun Yat-sen University, Guangzhou, Guangdong, China.

[2] Department of Orthodontics, Hospital of Stomatology, Sun Yat-sen University, Guangzhou, Guangdong, China.

[3] Guangdong Provincial Key Laboratory of Stomatology, Guanghua School of Stomatology, Sun Yat-sen University, Guangzhou, Guangdong, China.

E-mails:

Yuanyuan Chen: chenyy523@mail2.sysu.edu.cn; Guixin Cui: cuigx@mail2.sysu.edu.cn; Yuqi Cui: cuiyq5@mail2.sysu.edu.cn; Dongru Chen: chendr6@mail.sysu.edu.cn; Huancai Lin: linhc@mail.sysu.edu.cn

[*] Corresponding author:

Dongru Chen; Email: chendr6@mail.sysu.edu.cn

Huancai Lin; Email: linhc@mail.sysu.edu.cn

Tel: +86-20-8386-2560

Fax: +86-20-8382-2807

Materials and Methods

Virtual screening

We used MOE v2015.1001 for structure-based virtual screening. The X-ray structure of C123 segment was obtained from RCSB Protein Data Bank (PDB ID: 3QE5), where C3 was extracted. Specs library were used as a screening library. Firstly, all compounds were dealt with the Wash module in MOE, then all compounds were ranked according to London dG scoring with high throughput rigid docking, and top 10 K compounds were enrolled in the flexible docking with the aid of "induced fit" method. Next, the force field in AMBER12: EHT as well as implicit solvation model in Reaction Field (R-field) were utilized before docking. We optimized the protonation and hydrogen state of the C3 segment with LigX module and set the situation at the pH = 7 and temperature = 300 K. At last, flexible docking were conducted where the docked poses were firstly ranked based on London dG scoring, and a force field was refined on the top 10 poses with a rescoring of GBVI/WSA dG. Finally the top 99 hits of Specs were identified.

qRT-PCR For gene expression

qRT-PCR was utilized to quantify the gene expression level according to manufactures' instruction. Reaction mixture at the volume of 20 μL were depicted below: 10 μL SYBR green PCR master mix (Hieff® qPCR SYBR® Green Master Mix, Yeasen Biotech CO., Ltd, China), 0.8 μL for forward and reverse primers, 2 μL template DNA and 6.4 μL DEPC water. Real-time PCR were designated as follows: 95 °C for 300s, 40 repeats of 95 °C for 10 s, 60 °C for 20 s and 72 °C for 20 s. Sequences of primes were presented in Table S1.

Biofilm removal assays

The effects of D25 on *S. mutans* biofilms at distinct conditions were assessed. In brief, *S. mutans* were incubated anaerobically to form biofilms. After 4, 8, 16 and 24 h, the medium were aspirated and the biofilms were treated with D25 at the concentration of 6.25 μg/mL and incubated for another 24 h. Thereafter, the biofilms were fixed and measured biomass using crystal violet staining as described above. Meanwhile, mature biofilms formed after 24 h were treated with D25 at the concentration of 1.56 to 25 μg/mL as detailed in the text. The biomass was also evaluated by crystal violet staining as mentioned before. Statistical analysis of all data were performed with GraphPad Prism 7 (GraphPad Software, San Diego, USA) using Student's two-sample t-test. Significant differences were set at the 95% confident level.

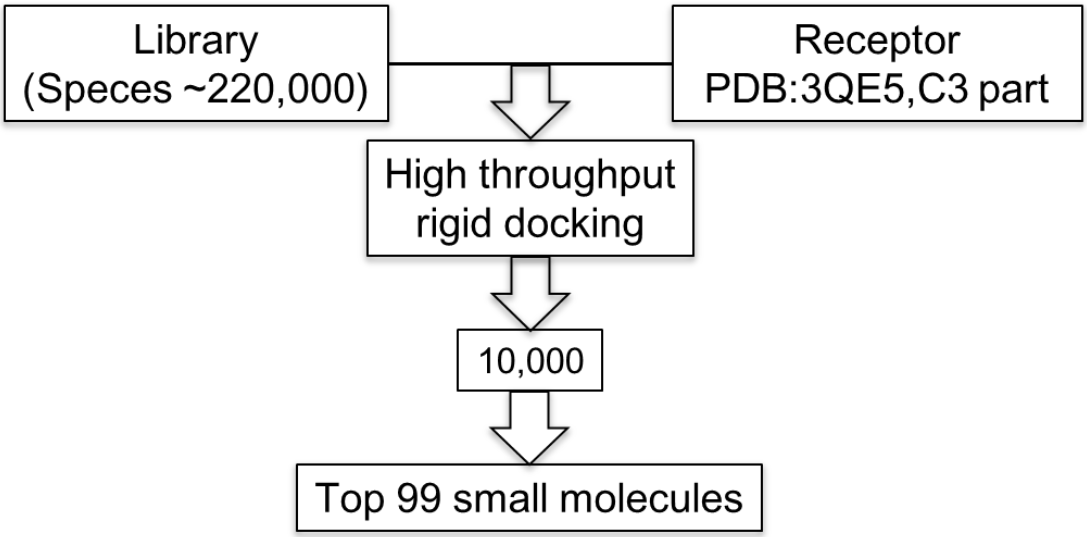

Figure S1 The flowchart of structure-based virtual screening.

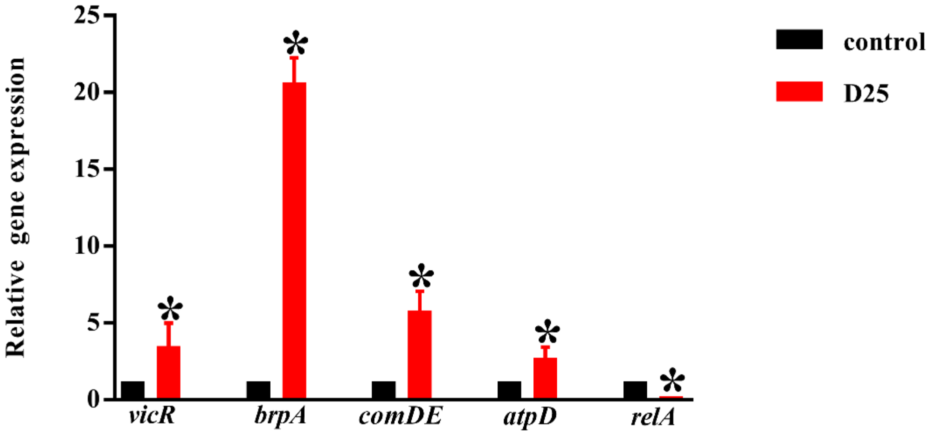

Figure S2 Effects of compound D25 on the expression of *vicR*, *brpA*, *comDE*, *atpD* and *relA* genes. The results from qRT-PCR were obtained with the $2^{-\Delta\Delta CT}$ method. All results were shown as mean ± standard deviations from three independent experiments. *, differences were statistically significant when compared with control group (P < 0.05).

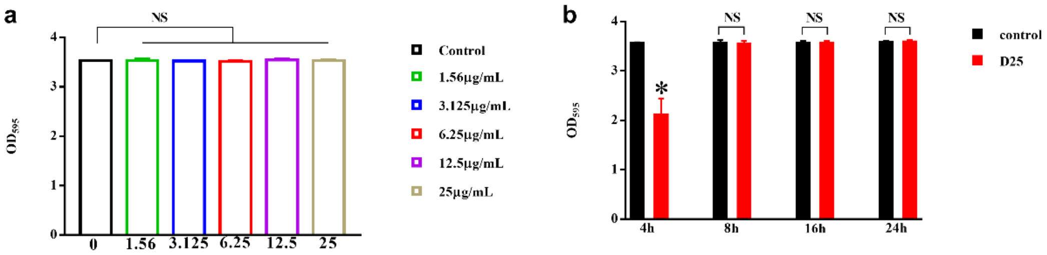

Figure S3 Effects of D25 on biofilms at different concentrations and time intervals. **a** *S. mutans* biofilms formed after 24 h were treated with different concentrations of D25. NS, differences were not statistically

significant when compared with control group. **b** Effects of D25 on *S. mutans* biofilms formed after 4, 8, 16 and 24 h. *, differences were statistically significant when compared with control group ($P < 0.05$). NS, differences were not statistically significant when compared with control group.

Table S1 Primers used in this study for qRT-PCR to quantify gene expression level.

Primes	Nucleotide Sequence (5'-3')	References
16s RNA-F	5'-CTTACCAGGTCTTGACATCCCG-3'	(Li et al. 2018)
16s RNA-R	5'-ACCCAACATCTCACGACACGAG-3'	
srtA-F	5'-GAAGCTTCCTGTAATTGGCG-3'	(Li et al. 2018)
srtA-R	5'-TTCATCGTTCCAGCACCATA-3'	
pacR-F	5'-GACTTTGGTAATGGTTATGCATCAA-3'	(Li et al. 2018)
pacR-R	5'-TTTGTATCAGCCGGATCAAGTG-3'	
vicR-F	5'-TGACACGATTACAGCCTTTGATG-3'	(He et al. 2019)
vicR-R	5'-CGTCTAGTTCTGGTAACATTAAGTCCAATA-3'	
brpA-F	5'-GGAGGAGCTGCATCAGGATTC-3'	(Zhao et al. 2020)
brpA-R	5'-AACTCCAGCACATCCAGCAAG-3'	
comDE-F	5'-ACAATTCCTTGAGTTCCATCCAAG-3'	(He et al. 2019)
comDE-R	5'-TGGTCTGCTGCCTGTTGC-3'	
atpD-F	5'-TGTTGATGGTCTGGGTGAAA-3'	(Xu et al. 2011)
atpD-R	5'-TTTGACGGTCTCCGATAACC-3'	
relA-F	5'-ACAAAAAGGGTATCGTCCGTACAT-3'	(He et al. 2019)
relA-R	5'-AATCACGCTTGGTATTGCTAATTG-3'	

Table S2 Top 99 small molecules selected by targeting C3 segment.

Name (Database ID)	Docking Score	accessible[a]	dissolved in DMSO[b]	code in this study[c]
AJ-030/12105382	-9.0334539			
AG-205/33005040	-8.7709694			
AO-763/14815004	-8.4502993	No		
AC-776/15493088	-8.4416676			

AK-968/12973943	-8.1781998			
AN-329/40922913	-8.1487646		Yes	D13
AN-329/40869136	-8.1423845		Yes	D14
AQ-405/42300267	-8.0910368		Yes	D25
AE-641/00366010	-8.0623646		Yes	D6
AG-205/33005003	-7.9195437		Yes	D1
AG-690/36946065	-7.7946362		Yes	D4
AO-295/15484020	-7.7111669		Yes	D12
AN-329/11378358	-7.7030687			
AH-487/40936667	-7.6935005		Yes	D10
AG-690/36760011	-7.4458237	No		
AP-828/40969990	-7.4327574			
AF-399/42017858	-7.3187318		Yes	D2
AK-968/41172133	-7.2762218		Yes	D3
AK-968/40337278	-7.2611494		Yes	D5
AK-968/41924813	-7.2485061			
AN-988/40787335	-7.2341418		Yes	D21
AJ-292/14241003	-7.1575971		Yes	D9
AG-690/33095035	-7.1439466			
AN-329/12244630	-7.1276588		Yes	D7
AK-968/15603038	-7.1146321			
AN-329/40614587	-7.0713944			
AG-690/13701210	-7.0665359			
AE-848/37236068	-7.0612454		Yes	D8
AG-690/33095033	-7.0505381			
AK-968/13029369	-7.0412726		Yes	D11
AN-329/11140252	-7.0232868			
AP-263/41946485	-7.0181203	No		
AO-022/43452516	-7.0050678		Yes	D31

AN-989/14465007	-6.9879174		
AN-989/15133071	-6.9779348		
AO-081/15570099	-6.9649482	Yes	D24
AG-670/41897787	-6.9485807	Yes	D27
AF-399/14944030	-6.9389381		
AN-329/12393005	-6.9308062	Yes	D19
AG-690/11241412	-6.9258189		
AK-968/37202032	-6.9115748		
AP-906/41639626	-6.8927941	Yes	D20
AM-814/41093933	-6.8766088	Yes	D15
AN-648/41666225	-6.8744097		
AK-918/41676778	-6.8597665		
AK-968/41021583	-6.8533864	Yes	D32
AG-205/32366022	-6.8522239	Yes	D29
AO-861/15351016	-6.842351	Yes	D22
AG-690/32517015	-6.8387337	Yes	D16
AJ-292/41944536	-6.8350224	Yes	D17
AN-655/14614055	-6.8202996		
AK-968/41926615	-6.809855		
AG-205/36953353	-6.793582	Yes	D23
AN-023/14771025	-6.7817311		
AE-842/31981057	-6.7753167	Yes	D26
AG-690/12889320	-6.7213387		
AS-871/43475574	-6.7142501		
AO-801/41077351	-6.7012362	Yes	D28
AN-655/13943034	-6.6954532	Yes	D18
AK-968/37156054	-6.6903319	Yes	D30
AK-968/41025568	-6.688437	Yes	D33
AP-006/41010231	-6.6830568		

AN-329/41434969	-6.6671605		Yes	D34
AK-968/11565149	-6.6249909			
AN-465/13570104	-6.6206021		Yes	D35
AF-962/32159049	-6.6044345			
AG-650/41069277	-6.6000171			
AK-777/11761035	-6.595283			
AP-653/41544882	-6.5851698			
AH-487/40716398	-6.580194		Yes	D36
AO-081/41715312	-6.5782266		Yes	D37
AT-057/43485784	-6.5480027		Yes	D38
AK-918/43446363	-6.5438714		Yes	D39
AN-584/43462284	-6.540699	No		
AE-848/42434927	-6.5384293		Yes	D40
AO-081/14336112	-6.5314841		Yes	D41
AG-690/40135004	-6.5241876			
AK-968/41923827	-6.5086575		Yes	D52
AS-871/43478157	-6.4983797		Yes	D55
AP-406/41885719	-6.4979715			
AG-690/11085024	-6.4913282		Yes	D42
AP-906/41647557	-6.4867191		Yes	D43
AK-968/15361557	-6.4785218		Yes	D44
AM-807/14146116	-6.468154		Yes	D45
AN-698/14990012	-6.4593225			
AO-022/43454978	-6.4592953			
AN-689/41741199	-6.456862			
AN-652/13064001	-6.4533682		Yes	D46
AN-919/40736967	-6.4520273		Yes	D47
AK-968/12385565	-6.444633		Yes	D48
AK-968/41924318	-6.4379086		Yes	D49

AG-670/36062016	-6.4365873		Yes	D50
AM-900/12897042	-6.4347148			
AN-465/43369895	-6.431859		Yes	D51
AH-034/32464023	-6.42485	No		
AK-820/13220141	-6.4085131			
AE-641/40789605	-6.4008918			
AK-968/41169804	-6.3960643		Yes	D54
AO-022/43453830	-6.3873978		Yes	D53

a: Small molecules were able to be purchased from Specs company with enough mass used in further study.

b: Small molecules were able to dissolved in DMSO to get 10 mg/mL stocking solution.

c: Small molecules were presented as specific codes in this article.

Reference

He Z, Huang Z, Jiang W, and Zhou W (2019) Antimicrobial activity of cinnamaldehyde on *Streptococcus mutans* biofilms**.** Front Microbiol 10. doi:10.3389/fmicb.2019.02241

Li B, Li X, Lin H, and Zhou Y (2018) Curcumin as a promising antibacterial agent: Effects on metabolism and biofilm formation in *S. mutans***.** Biomed Res Int 2018:4508709. doi:10.1155/2018/4508709

Xu X, Zhou XD, and Wu CD (2011) The tea catechin epigallocatechin gallate suppresses cariogenic virulence factors of *Streptococcus mutans***.** Antimicrob Agents Ch 55:1229-1236. doi:10.1128/AAC.01016-10

Zhao M, Qu Y, Liu J, Mai S, and Gu L (2020) A universal adhesive incorporating antimicrobial peptide nisin: Effects on *Streptococcus mutans* and saliva-derived multispecies biofilms**.** Odontology 108:376-385. doi:10.1007/s10266-019-00478-8
